# Physical Activity Measurement in People with Spinal Cord Injury: A Comparative Review of Different Questionnaires

**DOI:** 10.3390/jcm13226997

**Published:** 2024-11-20

**Authors:** Joan Úbeda-Colomer, Alex Castan

**Affiliations:** 1 Department of Physical Education and Sports, University of Valencia, 46010 Valencia, Spain; joan.ubeda-colomer@uv.es; 2 Institut Guttmann Neurorehabilitation Hospital, University Institute Attached to the Autonomous University of Barcelona, 08916 Badalona, Spain; 3 Institute for Health Science Research Germans Trias i Pujol (IGTP), 08916 Badalona, Spain; 4 Autonomous University of Barcelona (UAB), 08193 Cerdanyola del Vallés, Spain

**Keywords:** spinal cord injury, physical activity assessment, self-report questionnaires, validity, reliability

## Abstract

Physical activity (PA) provides great health benefits for people with spinal cord injury (SCI). Consequently, the design and implementation of PA interventions addressed to this population is needed. To rigorously evaluate these interventions, the use of valid and comprehensive PA measures is crucial. Since the suitability of PA assessment tools might differ among different populations, and considering that questionnaires are one of the most frequently used tools to quantify PA, the purpose of this comparative review was to examine nine questionnaires that have been used to assess PA in people with SCI. All the questionnaires were analyzed in depth in regard to three main dimensions: (1) SCI-specific development; (2) PA domains measured and PA intensity classification; and (3) reliability and validity. After careful consideration of the evidence available on all these aspects, it is suggested that the most suitable questionnaires to be used in PA research in the SCI population are the PARA-SCI and the LTPAQ-SCI[R]. To conclude, the strengths and limitations of these two questionnaires are discussed, and specific recommendations to SCI researchers and practitioners regarding the suitability, according to the context and characteristics, of the research/intervention are provided.

## 1. Introduction

According to the World Health Organization, 250,000 to 500,000 individuals worldwide experience a spinal cord injury (SCI) every year [1]. While a reliable global prevalence estimate is not available, the yearly incidence worldwide is estimated to range from 40 to 80 cases per million people [1]. SCI has consequences that reach beyond the nervous system, as it is a transformative event that impacts health [2], daily functioning [3] and psychosocial factors [4,5,6]. There is strong scientific evidence of the benefits of physical activity (PA) for people with SCI, such as improvements in muscle strength [7], metabolic health [8], cardiovascular functioning [9], functionality [10] and psychosocial variables such as quality of life [11], depressive and anxiety symptoms [12] and community integration [13]. However, less than a third of the SCI population is regularly active [14], and the majority of people with SCI usually report low levels of leisure-time physical activity (LTPA) [15,16]. In fact, individuals with SCI are generally regarded as being at the lower end of the physical activity spectrum, when compared to the general population or to those with other disabilities and chronic illnesses [17].

Given the great benefits that PA might bring to people living with SCI [18,19] and their high levels of inactivity, PA interventions that encourage physically active lifestyles among this population are needed. For these interventions to be effective, rigorous assessment and quantification of PA are crucial [20] and should align with the target population, the goals of the intervention and the context in which it occurs. For instance, as noted before, individuals with SCI typically do not engage in the minimum level of PA required to attain significant health benefits [15,21] and, when they are active, they are usually involved in low-intensity activities [22], which do not provide the same benefits as moderate- to vigorous-intensity PA. Therefore, in an intervention addressed to this population, it could be especially relevant to choose a PA measure which discriminates between mild, moderate and vigorous PA. Also, distinguishing between types of PA might be important in some cases, since this information is crucial for promoting health improvements through targeted programs or recommendations [23]. PA refers to any movement of the body caused by skeletal muscles that results in energy expenditure. This includes everyday activities, work tasks that involve physical effort, exercise for fitness, and both recreational and competitive sports [24]. However, all these activities can be categorized based on the context in which they occur and are typically classified into two distinct categories according to their type or purpose. On the one hand, lifestyle physical activity includes all the tasks that are part of a person’s daily routine, such as personal hygiene, household chores, work-related activities or sedentary leisure activities [25]. On the other hand, LTPA includes activities that involve physical effort and are chosen by individuals during their leisure time, such as playing sports, working out at a gym or going for a wheelchair outing [26]. Since LTPA has been shown to provide greater physiological, psychological and social benefits compared to lifestyle PA [27,28], the ability to discriminate between these two types when measuring PA could be relevant depending on the research or intervention purpose. Therefore, the selection of a valid and reliable PA measurement tool which aligns well with the intervention purpose becomes a relevant concern. There is a wide range of methods and techniques available for assessing PA. These include (a) self-reports, (b) movement sensors such as pedometers and accelerometers, (c) direct observation systems, (d) heart rate monitors and (e) indirect calorimetry and the doubly labeled water technique [29].

Among all these methods, self-report questionnaires have been the most extensively used in PA research given their multiple advantages. Questionnaires possess the characteristics of non-reactivity (i.e., they do not alter the behavior of the individual being surveyed), practicality (study costs are reasonable and they are convenient for participants), applicability (the instrument can be designed to fit the particular population in question) and accuracy (they are reliable and valid) [30]. For instance, they might be able to capture potentially important information regarding PA type (e.g., lifestyle PA or LTPA), while other methods such as accelerometer-based PA assessments report cumulative daily PA with no distinction. When it comes to cost, questionnaires and self-reported records are the least expensive options for assessing PA and are particularly beneficial in large-scale population or epidemiological research [31,32].

Many different questionnaires have been used to measure PA. However, not every questionnaire is suitable, nor do they possess the appropriate characteristics for every population and context. Since the “one-size-fits-all” approach could lead to a lack of accuracy and rigor in the evaluation of PA interventions, PA assessment tools should consider the characteristics of different population groups. Given the SCI population’s specific characteristics and particularities in relation to PA, this need becomes crucial in this group [31]. However, the validity and reliability of some PA measures that have been used in the SCI population have only been tested in the general population, ignoring the particularities and specific characteristics and needs of people living with SCI. In this regard, Ginis et al. [33] warned of an absence of data regarding the psychometric properties of the physical activity measures used in research involving individuals with SCI, and they made an urgent call to use measures that have been proven valid and reliable in this population. Addressing this call, the purpose of this comparative review is (a) to examine and compare the validity and reliability of different questionnaires that have been used to assess PA in people with SCI; and (b) to provide specific recommendations to SCI researchers and practitioners based on the strengths, limitations and suitability of the most valid and reliable questionnaires depending on the context and the research/intervention purpose.

## 2. An Overview of the Questionnaires

An exhaustive search was carried out in the most relevant scientific databases (Web of Science, Scopus, PubMed) to identify studies that used questionnaires to measure PA in the SCI population. To be included in the present review, the questionnaires had to meet two basic criteria: (1) they should have been used to measure PA in a sample of people with SCI or in a wider sample including people with SCI; and (2) they should be supported by, at least, a validation study analyzing their psychometric properties. Following these criteria, 9 questionnaires were selected. Table 1 summarizes their main characteristics. To analyze the properties of the questionnaires, studies examining the development, reliability and/or validity of each questionnaire in its original language were comprehensively reviewed. Studies on translations of the questionnaires were excluded from the in-depth analysis, since not all the questionnaires had versions in different languages.

*The Godin Leisure-Time Exercise Questionnaire (GLTEQ)*. This was developed for a healthy general population and consists of three questions that measure the average frequency and duration of light (minimal effort, no sweating), moderate (not tiring, light sweating) and strenuous (heart beats rapidly, sweating) PA lasting for more than 15 min during free time in a typical week. The scores are multiplied by weights and summed into an overall score (i.e., leisure-time physical activity [LTPA] score) that ranges between 0 and 119 metabolic equivalents of task (METs)/minutes of PA [34].

*The International Physical Activity Questionnaire—Long Form (IPAQ-LF).* This is the most scientifically reliable questionnaire used worldwide to assess PA in healthy populations [35]. It was originally designed to quantify PA in adults (age 18–65 years) for the cross-national monitoring of PA and inactivity across five domains, including work, household, leisure, transportation and sedentary time, and distinguishes between moderate and vigorous PA. It can be administered through telephone interviews or self-report. These versions include two different reference periods: either the “last 7 days” or a “typical week” of recalled physical activity. This questionnaire has also been used to measure PA in SCI populations [36]. This questionnaire categorizes individuals into three levels (low, medium, high) based on the estimated energy expenditure of each activity: vigorous (8 MET), moderate (4 MET) and walking (3.3 MET).

*The International Physical Activity Questionnaire—Short Form (IPAQ-SF).* This questionnaire is the reduced version of the IPAQ-LF. It provides information on the time spent walking, on vigorous- and moderate-intensity activity and on sedentary activity, with two recall periods: “last 7 days” or “usual week”. Also, it can be administered through telephone interviews or self-report [36].

*The International Physical Activity Questionnaire Adapted (IPAQ-D).* A framework was developed to adapt the IPAQ to be inclusive of people with disabilities. Changes to the instrument include using more inclusive PA examples, rephrasing “walking” to include use of a wheelchair, and rephrasing “sitting time” to reflect “sedentary time” along with an explanation of the definition of sedentary time [37].

*The Physical Activity Scale for Individuals with Physical Disabilities (PASIPD).* This was created by adapting the Physical Activity Scale for the Elderly (PASE) [38] for application in epidemiological research on PA, health and functionality, particularly in individuals with physical disabilities. The development of this tool involved a study group comprising individuals with various disabilities, including post-polio syndrome, SCI, cerebral palsy, amputations, muscular dystrophies, spina bifida, and visual and hearing impairments, as well as individuals with other conditions such as epilepsy, hemophilia and diabetes [39]. It is a brief questionnaire consisting of 13 items: 6 related to leisure activities, 6 to household tasks and 1 to paid employment. It can be administered in person, via email or over the phone, with the purpose of gathering PA data for the preceding 7 days. The scale is scored to produce a composite PASIPD score, which is calculated by multiplying the average hours per day by a MET value. This MET value corresponds to the intensity of the activity, and the result is ultimately expressed as MET-hours per day to represent PA patterns [39].

*The Physical Activity Inventory-SCI (PAI-SCI).* The PAI-SCI is a PA questionnaire designed specifically for individuals with SCI. It is based on the PASIPD questionnaire, with the original items modified to better suit the unique needs of people with SCI [40]. It includes 14 items, which refer to the 7 days leading up to the administration of the questionnaire, in which the participant reports the number of days in the previous week that they engaged in specific activities. Participants are requested to report the number of minutes or hours spent on a specific activity. The total scores are determined by summing the daily or average hours at a given intensity, which are then multiplied by a previously determined equivalent metabolic value.

*The Physical Activity Recall Assessment for People with Spinal Cord Injury (PARA-SCI).* This was created to assess the type, frequency, duration and intensity of PA in individuals with SCI who primarily use a wheelchair for mobility. The tool is applicable to people with either paraplegia or tetraplegia and is intended to measure three categories of PA: LTPA, lifestyle activity and cumulative activity (which combines both LTPA and lifestyle activity). The development of the PARA-SCI involved creating (a) a semi-structured interview protocol for collecting PA information and (b) a PA Intensity Classification System. The participant is asked to recall the activities performed in the three days leading up to the interview and categorize them based on the PA Intensity Classification System (which they will have reviewed with the interviewer beforehand). Activities are classified into one of the following intensity levels: “Nothing at all”, “Mild”, “Moderate” or “Heavy”. This process results in the calculation of minutes per day and the total for both LTPA and lifestyle activity for each intensity category [41].

*The Leisure Time Physical Activity Questionnaire for People with SCI (LTPAQ-SCI)*. The LTPAQ-SCI is a self-report questionnaire created specifically for individuals with SCI. It uses the same PA Intensity Classification System as the PARA-SCI, but in contrast to the PARA-SCI, it exclusively measures the duration of LTPA in minutes [26]. Its structure is based on the GLTEQ [34] and the IPAQ-SF [35], reporting activity in the preceding 7 days. A revised version of the LTPAQ-SCI, the LTPAQ-SCI[R], has recently been developed. Like the LTPAQ-SCI, this questionnaire records the number of days and minutes spent on mild-, moderate- and vigorous-intensity aerobic LTPA. However, it introduces one additional item to track the number of days and minutes dedicated to moderate to vigorous strength-training LTPA over the preceding seven days.

*The Leisure Time Physical Activity Questionnaire for People with Disability (LTPAQ-D).* This is an adapted version of the LTPAQ-SCI[R] to be used in populations with different kinds of disability. Respondents report on LTPA activity in the preceding 7 days, and it can be self-administered or administered by interview [42]. Like LTPAQ-SCI[R], it reports the average number of minutes per day (mild, moderate, heavy, total) for aerobic LTPA and the average number of minutes per day for moderate to vigorous strength training.

## 3. Results

The questionnaires selected were analyzed in depth in terms of their SCI-specific development or features, the PA domains measured and the PA intensity classification they provide, as well as their validity and reliability.

### 3.1. The Specificity, Physical Activity Domains and Intensity Classification of the Questionnaires

Of all the questionnaires included in this review, only three (the PAI-SCI, the PARA-SCI and the LTPAQ-SCI) were specifically tailored to a SCI population. The IPAQ’s forms and the GLTEQ were developed for a healthy general population. The PASIPD and the LTPAQ-D were developed for people with disabilities.

As described above, PA can be classified as LTPA and lifestyle activity. As shown in Table 1, of the questionnaires reviewed, the PASIPD, the PAI-SCI and the different forms of the IPAQ measure total PA, with no distinction between these two types of activity. In contrast, the GLTEQ, the LTPAQ-D and the LTPAQ-SCI only measure LTPA. The only questionnaire that can quantify PA according to its dimension is the PARA-SCI, classifying PA in terms of LTPA or lifestyle PA. However, the approach used in the LTPAQ-SCI [R] and the LTPAQ-D is promising, as they separate LTPA into aerobic LTPA and strength-training LTPA, thus allowing for an examination of if respondents meet the SCI exercise guidelines.

As shown in Table 1, the PASIPD and the PAI-SCI do not classify PA according to intensity. IPAQ’s forms classify it as moderate or vigorous. The GLTEQ, the PARA-SCI, the LTPAQ-SCI and the LTPAQ-D classify it as mild, moderate and vigorous.

### 3.2. The Reliability of the Questionnaires

Reliability refers to the extent to which measurements can be consistently replicated. Prior to being used in research or clinical settings, the reliability of any assessment tool must be established. This reflects not only the level of correlation between measurements but also the degree of agreement among them [43]. Specifically, test–retest reliability is commonly assessed, which measures the consistency of an instrument’s results when applied to the same subject under identical conditions at different times.

As shown in Table 2, the test–retest reliability of the nine reviewed questionnaires has been studied. For all of them, a second administration of the questionnaire was carried out after seven days, except for the IPAQ-D and the GLTEQ, for which a second assessment was carried out after three days and two weeks, respectively. In addition, for the LTPAQ-D, a third complementary administration was carried out after 2 days. The questionnaires with the highest test–retest reliability were the PARA-SCI, the LTPAQ-SCI, the LTPAQ-D and the IPAQ-LF. In contrast, the questionnaire with the lowest reliability values was the PAI-SCI.

### 3.3. The Validity of the Questionnaires

As shown in Table 3, validity was assessed in all the instruments, but only the PASIPD, the PAI-SCI, the PARA-SCI and the LTPAQ-SCI were tested in the SCI population. The GLTEQ, the various forms of the IPAQ, and the LTPAQ-D have not been tested in this population. The PARA-SCI is the questionnaire that accumulates the highest level of evidence of validity in assessing LTPA. In contrast, the PASIPD is the questionnaire whose use in the SCI population is most compromised.

## 4. Discussion

The main purpose of this comparative review was twofold. On the one hand, it aimed to provide an in-depth analysis of the questionnaires that have been used to assess PA in people with SCI. On the other hand, it intended to offer specific recommendations to SCI researchers and practitioners on the strengths, limitations and suitability of the most valid and reliable questionnaires depending on the context and the research/intervention focus. Addressing this purpose, the results of the analysis of the questionnaires are discussed in regard to three different dimensions: SCI-specific development, PA domains and PA intensity classification, and reliability and validity. Finally, a section discussing the practical implications of this review is provided.

### 4.1. SCI-Specific Development

Only three of the questionnaires included in this review—the PAI-SCI, the PARA-SCI and the LTPAQ-SCI—were explicitly designed for individuals with SCI. The GLTEQ and the IPAQ’s long and short forms were developed for a healthy population. The IPAQ-D, the PASIPD and the LTPAQ-D were developed for people with disabilities. As mentioned before, the use of PA assessment tools only validated in a general healthy population might lead to mistaken conclusions in the SCI population [55]. Several studies that have employed the IPAQ-LF or the IPAQ-SF to evaluate PA in individuals with disabilities have found that this tool may not be fully applicable to people with specific health conditions, such as those with SCI [56,57,58]. Some IPAQ items provide activity examples, such as walking or climbing, that are not typical or characteristic of the SCI population and could thus be confusing for the respondent. Regarding the PASIPD, its psychometric characteristics have been tested in a very heterogeneous disability population (e.g., persons with cerebral palsy, meningomyelocele or sensory impairments) whose characteristics differ greatly from the population with SCI [39,48]. Furthermore, both the PASIPD and the PAI-SCI assess PA in terms of metabolic equivalents (METs) using data from individuals without disabilities [59], which may not be appropriate or valid for populations with disabilities [60]. The PARA-SCI is the only questionnaire that incorporated the input of individuals with SCI during its development [41] and its PA Intensity Classification System, subsequently adopted by the LTPAQ-SCI, was specifically developed for the SCI population.

This is crucial, as any analysis of the relationship between PA and disease outcomes should rely on accurate data regarding the components of energy expenditure that represent the largest portion of total energy expenditure in the target population [30]. Laporte et al. [32] highlighted this point, noting that one source of variability in accurately characterizing PA is the precision of intensity attribution. Selecting the most appropriate questionnaire for a specific population and developing a specific energy expenditure prediction formula are thus crucial, especially in populations with neurological or physical problems [31]. Due to the physiological consequences of SCI, the assessment of PA intensity should be specific to the group. This specificity as well as the involvement of people with SCI in the development process of the questionnaire are strengths of the PARA-SCI [61], subsequently inherited by the LTPAQ-SCI and the LTPAQ-SCI[R].

### 4.2. PA Intensity Classification and PA Domains

In terms of classification of the different intensities of PA, the GLTEQ, the PARA-SCI, the LTPAQ-SCI and the LTPAQ-D can distinguish between mild, moderate and vigorous PA. In this regard, as noted in Section 4.1, the authors of the PARA-SCI undertook a systematic process to develop SCI-specific definitions of mild, moderate and heavy PA [41], which were also adopted by the LTPAQ-SCI, the LTPAQ-SCI[R] and the LTPAQ-D. This is a crucial aspect because PA intensity is essential in PA guidelines for adults with SCI. Evidence strongly supports that engaging in moderate to vigorous PA is crucial for obtaining substantial health benefits. To improve cardiorespiratory fitness and muscle strength, adults with SCI should engage in at least 20 min of moderate- to vigorous-intensity aerobic exercise twice a week, along with three sets of strength-training exercises for each major muscle group at a moderate to vigorous intensity, also twice a week. For cardiometabolic health benefits, adults with SCI are advised to engage in at least 30 min of moderate- to vigorous-intensity aerobic exercise three times per week [62]. Thus, the only questionnaires that can be adapted to the assessment of these PA guidelines are the PARA-SCI, the LTPAQ-SCI[R] and the LTPAQ-D. The first questionnaire can be adapted because the interview can be used to specifically collect data for all types of activities whose assessment is of interest, and the other two because their design has already been focused on collecting data for these two activities: aerobic exercise and strength training. Therefore, having assessment tools that classify PA according to its intensity is crucial for developing and assessing programs that achieve PA guidelines and recommendations for the SCI population.

Regarding the PA domains captured by the questionnaires, the PAI-SCI, the PASIPD and the IPAQ’s forms capture total PA, with no distinction between lifestyle PA and LTPA. The GLTEQ, the LTPAQ-SCI, the LTPAQ-SCI[R] and the LTPAQ-D only measure LTPA, while the PARA-SCI is the only questionnaire which can quantify lifestyle PA, LTPA and total PA. However, it should be noted that most research in the SCI population has focused on LTPA, given that most activities of daily living are generally not performed at an intensity or duration that would provide health or fitness benefits [28]. Since participation in regular LTPA is particularly relevant for the SCI population, tools that can quantify this PA domain are essential.

### 4.3. Reliability and Validity

Regarding the test–retest reliability of the questionnaires reviewed, this varies between the different studies. However, in general, the results suggest that all of them provide stable information, except for the PAI-SCI. In addition, some of the questionnaires have been translated and culturally adapted to other languages and contexts, testing their reliability as well. For instance, the PASIPD has been translated into Malay and adapted for people with SCI. This version showed acceptable test–retest reliability (intraclass correlation = 0.87) [63]. The PARA-SCI has also been translated and adapted to different languages and cultural contexts, showing good test–retest reliability [64,65,66,67]. In addition, inter-rater reliability, which represents the variability between two or more raters evaluating the same group of individuals, was also studied for the Spanish and Thai translations of the PARA-SCI [64,67]. Inter-rater reliability results of the ICC of three PA categories (LTPA, lifestyle and total) at the three intensity levels measured showed that this questionnaire, despite the complexity of interview administration and possible recall bias, is a reliable tool when administered by different people.

In relation to the validity of the questionnaires, only the IPAQ-LF, the PASIPD and the PARA-SCI compared the questionnaire measures to the doubly labeled water method, which is the gold standard for 24 h energy expenditure [68]. PA energy expenditure from doubly labeled water (in kJ) was significantly associated with the IPAQ-LF and the PARA-SCI (R2 = 0.50, *p* = 0.005) but not with the PASIPD [46,49]. However, since the use of doubly labeled water is complex and expensive, and given that its validity could be lower in individuals with health conditions that affect total body water content, another strategy to assess the validity of questionnaires is comparing them against accelerometers. For instance, the validity of the long and short versions of the IPAQ was assessed by comparison with accelerometers, finding a fair to moderate agreement between the two measures in a sample of 744 healthy adults [44]. The PASIPD was also tested by comparison with accelerometers, obtaining a criterion validity Spearman correlation of 0.30 in a sample of 45 participants. However, these were non-wheelchair-dependent people diagnosed with stroke, chronic pain, whiplash, and neurological, orthopedic or back disorders. Only one participant in this sample had an SCI [45]. In addition, de Grot et al. [47] examined the construct validity of the PASIPD against measures of physical fitness, finding divergent validity in a heterogeneous sample of 139 people with SCI living in the community. In contrast, the validity of the PARA-SCI has been confirmed by the positive correlation between aerobic fitness and muscle strength measures with LTPA and cumulative PA scores. Additionally, analyses of extreme groups have offered further support for its validity by highlighting differences between groups in the LTPA category [50]. Although the original version of the PARA-SCI has not been tested with accelerometers, studies of the PARA-SCI in other languages have added additional validity by obtaining a strong level of correlation between accelerometer data and PARA-SCI interviews for 3-day MVPA and total PA [55,62]. Regarding the LTPAQ-D and the LTPAQ-SCI, they have not been validated by comparison with accelerometer-measured PA. However, the LTPAQ-SCI showed that minutes per week of mild-, moderate- and heavy-intensity LTPA and total LTPA were all positively correlated with VO2peak. Also, correlations between LTPAQ-SCI variables and POpeak were positive but small [53]. Similarly, LTPAQ-D measures have shown medium to large correlations with other self-report measures of aerobic and strength-training PA, as well as moderate partial correlations with VO2peak and maximal strength, respectively [54]. Also, the GLTEQ showed strong individual correlations between subjective and objective data related to the VO2 max percentile of reported strenuous exercise (r = 0.38; *p* < 0.001) and to the BF percentile of self-ratings of sweat-inducing exercise (r = 0.21; *p* < 0.001).

Previous research has also investigated the psychometric properties of some of these questionnaires in similar populations. For instance, Lankhorst et al. [68] conducted a systematic review to assess the validity and reliability of self-report instruments for measuring physical activity in individuals who use wheelchairs. Their review included the PASIPD, the PAI-SCI, the PARA-SCI and the LTPAQ-SCI. They concluded that the PARA-SCI is the most appropriate tool for evaluating physical activity in both people with spinal cord injuries (SCIs) and wheelchair users, although they pointed out the lack of supporting data on its internal consistency. It is worth mentioning, however, that the PARA-SCI is a multidimensional measure, encompassing frequency, intensity, duration and type of activity, and that not all these dimensions need to be correlated with each other [69]. Also, Nightingale et al. [60] evaluated four questionnaires to predict PA and energy expenditure in individuals who primarily use wheelchairs for mobility: the Physical Activity Disability Survey (PADS), the PASIPD, the PARA-SCI and the LTPAQ-SCI. Their findings indicated that the PARA-SCI exhibited the strongest criterion validity and reliability. Tanhoffer et al. [49] compared the PASIPD and PARA-SCI as methods for assessing energy expenditure and PA in individuals with SCI. They determined that the PARA-SCI is the most accurate questionnaire for predicting energy expenditure resulting from PA in wheelchair users with SCI. In addition, Cervantes and Porreta [29] reviewed 27 studies that reported on the validity and reliability of instruments used to measure physical activity in individuals with disabilities. Of these, 13 studies utilized self-reported questionnaires as the tool for assessing physical activity. However, only two of these studies focused on individuals with spinal cord injuries (SCIs) when evaluating the validity and reliability of the instrument. These two studies were those related to the development [41] and validity [50] of the PARA-SCI.

### 4.4. Implications for Researchers and Practitioners

After careful consideration of all the dimensions analyzed in this review, we suggest that the most suitable questionnaires to be used in PA research in the SCI population are the PARA-SCI and the LTPAQ-SCI, especially the reviewed version (LTPAQ-SCI[R]). However, to help with the decision of when to use one or another, we provide some practical and specific considerations.

On the one hand, the PARA-SCI is the most complete PA measure, since it captures both LTPA—such as gym exercise or leisure sports—and lifestyle PA—such as hygiene, caring for others or housework. The PARA-SCI also allows one to distinguish between specific activities, such as minutes of transfers, minutes of caring for others or minutes of work in the case of lifestyle PA, and minutes of wheeling or recreational sports in the LTPA domain. However, it does not completely align with the SCI exercise guidelines, since these are based on weekly activity and the PARA-SCI collects data from the preceding 3 days. In addition, interview administration is more complex, and it is way more time-consuming than the LTPAQ-SCI or the LTPAQ-SCI[R], since it takes 30 min on average to complete, and some active respondents might need more than 45 min. In addition, the interviewer must complete the recording form during the interview, and when the interview is over, the scoring form must be completed. As indicated above, it is a time-consuming process, which may also initially appear difficult. However, for clinicians and researchers interested in its use, manuals in English and Spanish that provide all the necessary material and simple instructions for conducting systematic and simple interviews are available [70,71]. Therefore, we would recommend the PARA-SCI if the following criteria are met: (1) an in-depth analysis of both LTPA and lifestyle PA is essential to the intervention/research; (2) the number of participants is not massive and there is at least one trained interviewer fully involved with the project for the data collection process; and (3) response burden is not a main concern given the specific characteristics of the intervention/research.

On the other hand, the LTPAQ-SCI[R] measures the weekly minutes of mild-, moderate- and heavy-intensity leisure-time aerobic exercise, as well as the weekly minutes of moderate to vigorous strength-training. It thus allows for measurements of compliance with the SCI exercise guidelines. However, it does not capture lifestyle activity, nor does it allow one to distinguish between different LTPA types, such as different sports, gym exercise or wheeling. In exchange, the LTPAQ-SCI[R] takes just 5 min to complete and, given its simplicity compared to the PARA-SCI, it can be self-administered. We would thus recommend the LTPAQ-SCI[R] if the following criteria are met: (1) the aim is conducting a large-scale population-based study or an intervention involving a high number of participants; (2) the study/intervention is focused on LTPA and/or compliance with the SCI exercise guidelines; and (3) there is a need for an easy and brief administration process that reduces participants’ burden.

In addition, it should be highlighted that the LTPAQ-D might be the best choice when the study/intervention involves people with SCI as a part of a larger sample of people with disabilities, since it has all the strengths of the LTPAQ-SCI[R], while the small adaptations performed to be inclusive of different kinds of disability have proven to be reliable and valid.

## 5. Conclusions

The present review has examined in depth the characteristics and psychometric properties of different questionnaires that have been used to measure PA in people with SCI, highlighting some of their strengths and limitations. It thus provides a comprehensive analysis of these measures that could be of great interest for SCI researchers and practitioners in making informed choices on which PA measure to use for their projects and interventions.

After thoroughly analyzing the characteristics of the questionnaires and their psychometric properties, we conclude that the PARA-SCI and the LTPAQ-SCI[R] are the most appropriate for use in PA research in the SCI population. Also, we recommend the use of the LTPAQ-D when the study includes people with SCI as a part of a wider sample of people with disabilities.

However, the cultural context and the language in which the questionnaire is to be used should also be considered. The PARA-SCI and the LTPAQ-SCI[R] were originally developed in English. Currently, only the PARA-SCI has been translated, culturally adapted and validated in multiple languages and contexts worldwide. The LTPAQ-SCI was validated in Canadian French as well in a sample of people with different disabilities, but this was the version which does not include a measure of strength training, not the revised version. We thus invite researchers from non-English speaking countries to translate and develop culturally adapted versions of the LTPAQ-SCI[R] and the LTPAQ-D.

## Figures and Tables

**Table 1:** Main characteristics of each PA assessment questionnaire.

Name	Pop.	Domains	Number of Questions	Adm. Mode	Compl. Time	Non-English Versions	Recall Period	PA Intensity Class.
GLTEQ	Healthy general pop.	(a) Leisure exercise	4 items/ questions	Self-adm.	5 min	yes	7 days	mild, moderate, strenuous
IPAQ-LF	Healthy general pop.	(a) Job-related PA; (b) Transportation PA; (c) Housework, house maintenance and caring for family PA; (d) Recreation sport and leisure PA; and (e) Sedentary time	27 items/ questions	Self-adm.	10 min	yes	7 days	moderate, vigorous
IPAQ-SF	Healthy general pop.	(a) Sports and leisure PA; (b) Walking; and (c) Sedentary activity	7 items/ questions	Self-adm.	5 min	yes	7 days	moderate, vigorous
IPAQ-D	Disab.	(a) Sports and leisure PA; (b) Walking; and (c) Sedentary activity	7 items/questions	Self-adm.	5 min	no	7 days	moderate, vigorous
PASIPD	Disab.	(a) Leisure-time PA; (b) Household activity; and (c) Occupational PA	13 items/questions	Self-adm.	5 min	yes	7 days	no
PAI-SCI	SCI	(a) Exercise general; (b) Activity/self-care; (c) Household; and (d) Outdoor/gardening	14 items/questions	Self-adm.	5 min	no	7 days	no
PARA-SCI	SCI	(a) LTPA; and (b) Lifestyle PA	Semi-structured interview protocol	Interview (face to face or phone)	30 min	yes	3 days	mild, moderate, heavy
LTPAQ-SCI	SCI	(a) LTPA	6 items/questions	Self-adm.	5 min	yes	7 days	mild, moderate, heavy
LTPAQ-D	Disab.	(a) Aerobic LTPA; and (b) Strength-training LTPA	9 items/questions	Self-adm.	5 min	yes	7 days	mild, moderate, heavy

**Table 2:** Reliability of the PA assessment questionnaires.

Instrument	Study	Participants	Test–Retest Time	Results
GLTEQ	Godin and Shephard 1985 [34]	53	2 weeks	Safrit reliability coefficients were 0.94, 0.46, 0.48 and 0.80 for strenuous, moderate, light and sweat-inducing exercise
IPAQ-SF	Craig et al. 2003 [44]	1974	7 days	Spearman correlation coefficients ranged from 0.32 to 0.88
IPAQ-LF	Craig et al. 2003 [44]	1880	7 days	Spearman correlation coefficients ranged from 0.46 to 0.96
IPAQ-D	Clina et al. 2024 [37]	102	3 days	ICC ranged from 0.532 to 0.835 for the eight measures of activity in people with disability
PASIPD	van der Ploeg et al. 2007 [45]	45	7 days	Spearman correlation coefficient was 0.77
PAI-SCI	Butler et al. 2008 [40]	43	7 days	ICC ranged from 0.28 to 0.59
PARA-SCI	Ginis et al. 2005 [41]	102	7 days	ICC ranged from 0.65 to 0.80 for total PA; ICC ranged from 0.63 to 0.91 for LTPA; ICC ranged from 0.56 to 0.80 for lifestyle activity
LTPAQ-SCI	Martin Ginis et al. 2012 [26]	35	7 days	ICC was 0.83 for total activity; ICC was 0.74 for mild activity; ICC was 0.62 for moderate activity; ICC was 0.93 for heavy activity
LTPAQ-D	Cummings et al. 2019 [42]	37	2 days/7 days	ICC ranged from 0.72 to 0.92/from 0.54 to 0.75

**Table 3:** Validity and related measurement properties of the PA assessment questionnaires.

Instrument	Study	Participants	Sample	Validity Assessment
GLTEQ	Godin and Shephard 1985 [34]	306	General population	There were correlations between subjective and objective data related to the VO2 max percentile of reported strenuous exercise (r = 0.38; *p* < 0.001) and to the BF percentile of self-ratings of sweat-inducing exercise (r = 0.21; *p* < 0.001).
IPAQ-SF	Craig et al. 2003 [44]	781	General population	There was moderate agreement between IPAQ data against accelerometers (pooled 0.30, 95% CI 0.23–0.36).
IPAQ-LF	Craig et al. 2003 [44]	744	General population	There was moderate agreement between IPAQ data against accelerometers (pooled 0.33, 95% CI 0.26–0.39).
	Hagströmer et al. 2006 [35]	46	General population	There were strong positive relationships between activity monitor data and the IPAQ data for total PA (r ¼ 0.55, *p* < 0.001) and vigorous PA (r ¼ 0.71, *p* < 0.001). Calculated MET-h day21 from a PA logbook was significantly correlated with MET-h day21 from the IPAQ (r ¼ 0.67, *p* < 0.001).
	Hagströmer et al. 2006 [35]	46	General population	There were weak correlations between IPAQ data and both aerobic fitness (r ¼ 0.21, *p* ¼ 0.051) and body mass index (r ¼ 0.25, *p* ¼ 0.009).
	Madisson et al. 2007 [46]	36	General population	There were moderate correlations between IPAQ and doubly labeled water energy expenditure (rs = 0.31).
IPAQ-D	Clina et al. 2024 [37]	62	People with and without disability	There were good correlation coefficients (0.727; 95% CI, 0.579–.829; *p* < 0.001). Construct validity assessment yielded a moderate coefficient (0.406; 95% CI, 0.166–0.596; *p*= 0.001).
PASIPD	Washburn et al. 2002 [39]	372	People with disability	Exploratory factor analysis revealed 5 latent factors: home repair and lawn and garden, housework, vigorous sport and recreation, light sport and recreation, and occupation and transportation. Cronbach coefficients ranged from 0.37 to 0.65, indicating low-to-moderate internal consistency within factors. Those who reported being “active/highly active” had higher total and subcategory scores compared with those “not active at all” (all *p* < 0.05).
	van der Ploeg et al. 2007 [45]	45	People with disability (non-wheelchair)	The criterion validity Spearman correlation was 0.30 when compared to accelerometers.
	de Groot et al. 2010 [47]	139	SCI population	Persons with tetraplegia had significantly lower PASIPD scores than those with paraplegia (*p* < 0.02). Persons with longer TSI had lower PASIPD scores than persons with shorter TSI (*p* < 0.03).
	de Groot et al. 2010 [47]	139	SCI population	PASIPD scores showed moderate correlations with related activities (0.36–0.51, *p* < 0.01) and weak to moderate correlations with fitness parameters (0.25–0.36, *p* < 0.05). In a fairly homogeneous group of people with SCI, 1 year after in-patient rehabilitation, the PASIPD showed weak-to-moderate relationships with activity and fitness parameters. There seems to be a limited association between self-reported activity level and fitness in people with SCI.
	van den Berg-Emons et al. 2011 [48]	124	Ambulatory and non-ambulatory persons with cerebral palsy, meningomyelocele or spinal cord injury	Significant Spearman correlation coefficients between the PASIPD and activity monitor outcome measures ranged from 0.22 to 0.37. The PASIPD overestimated the duration of physical activity measured using the activity monitor (mean ± SD, 3.9 ± 2.9 vs. 15 ± 0.9 h/d; *p* < 0.01). A significant correlation (*p* = −0.4; *p* > 0.01) was found between the average number of hours of physical activity per day measured using the 2 methods and the difference in hours between methods. This indicates larger overestimation for people with higher activity levels.
	Tanhoffer et al. 2012 [49]	14	SCI population	The PASIPD was not significantly associated with doubly labeled water PAEE, heart rate monitoring or SWA.
PAI-SCI	Butler et al. 2008 [40]	43	SCI population	Internal consistency was measured using Cronbach’s alpha coefficient [light household activity (α = 0.90), outdoor work/gardening (α = 0.82), exercise (α = 0.81) and general activity/self-care (α = 0.35)].
PARA-SCI	Ginis et al. 2005 [41]	14	SCI population	PARA-SCI intensities (moderate, heavy and total) and indirect calorimetry measures ranged from r = 0.63 to 0.88; mild-intensity cumulative activity was r = 0.27 but not significant.
	Latimer et al. 2006 [50]	73	SCI population	LTPA and cumulative activity were positively correlated with measures of aerobic fitness (r = 0.26–0.35) and muscle strength (r = 0.22–0.36); lifestyle activity was not generally associated with aerobic or muscle strength.
	Latimer et al. 2006 [50]	158	SCI population	Men and younger participants reported more total LTPA compared with women and older participants; people who were employed or attending school accumulated more minutes of mild-intensity lifestyle activity than those who were not.
	Tanhoffer et al. 2012 [49]	14	SCI population	The PARA-SCI was significantly associated with doubly labeled water PAEE (in kJ) (R2 = 0.50, *p* = 0.005), but not with heart rate monitoring and SWA.
	Claridge et al. 2015 [51]	42	Adults with cerebral palsy	The PARA-SCI correlated significantly with accelerometer-measured min. of MVPA per day (r = 0.396; *p* = 0.014) and per hour of monitoring time (r = 0.356; *p* = 0.027).
	Ma et al. 2020 [52]	19	SCI population	Total and wheeled moderate–vigorous PA measured by an accelerometer and the PARA-SCI showed low agreement at the individual level.
LTPAQ-SCI	Martin Ginis et al. 2012 [26]	103	SCI population who used a wheelchair as the primary mode of mobility	All correlations between the PARA-SCI and LTPAQ-SCI measures of LTPA were positive and statistically significant (*p* < 0.05). The strongest correlation was between the measures of heavy LTPA (*p* = 0.54), followed by the measures of total (*p* = 0.46) and moderate LTPA (*p* = 0.43). The weakest correlation was between the measures of mild-intensity LTPA (*p* = 0.27)
	Martin Ginis et al. 2021 [53]	39	SCI population	Minutes per week of mild-, moderate- and heavy-intensity LTPA and total LTPA were all positively correlated with VO2peak. The correlation between min/week of mild intensity LTPA and VO2peak was small–medium (r = 0.231, *p* = 0.079), while all other correlations were medium–large (rs ranged from 0.276 to 0.443, *p* < 0.05). Correlations between LTPAQ-SCI variables and POpeak were positive but small (rs ranged from 0.087 to 0.193, *p* > 0.05), except for a medium-sized correlation between heavy-intensity LTPA and POpeak (r = 0.294, *p* = 0.035).
LTPAQ-D	Gee et al. 2024 [54]	27	People with physical or sensory disabilities	LTPAQ-D measures of aerobic LTPA and strength training shared medium to large correlations with other self-report measures of aerobic PA and strength training (r = 0.458–0.942, *p* < 0.01). After controlling for age, aerobic LTPA and MVPA shared moderate partial correlations with VO2peak (r = 0.341 and 0.356, respectively). Min/week of strength training was associated with predicted maximal strength on the chest press (r = 0.621, *p* = 0.009).

## Data Availability

The corresponding author can be contacted for further information.
